# Meat quality assessment at different slaughter weights of broilers sold in the retail market of Dhaka City, Bangladesh: An integrated approach

**DOI:** 10.5455/javar.2026.m1014

**Published:** 2026-03-09

**Authors:** Md. Saiful Islam, Md. Jahidul Islam, Zannatul Naim, Rahat Nahiyan Ramim, Jianfeng He, Md. Asaduzzaman, Md. Najmul Haque, Md. Ismail, Masud Bin Tuhin, Md. Arafat Hossain, Muhammad Ashad Kabir

**Affiliations:** 1 Department of Animal Production and Management, Faculty of Animal Science & Veterinary Medicine, Sher-e-Bangla Agricultural University, Dhaka 1207, Bangladesh; 2 Department of Animal Science, University of Connecticut, Storrs, CT 06269, USA; 3 Faculty of Animal Science & Veterinary Medicine, Sher-e-Bangla Agricultural University, Dhaka 1207, Bangladesh; 4 Inner Mongolia Academy of Agricultural and Animal Husbandry Sciences, Hohhot 010031, China; 5 Department of Dairy Science, Faculty of Animal Science & Veterinary Medicine, Sher-e-Bangla Agricultural University, Dhaka 1207, Bangladesh; 6 School of Computing, Mathematics and Engineering, Charles Sturt University, Bathurst, NSW 2795, Australia

**Keywords:** Broiler, nutritional profiles, physical properties, sensory evaluation, slaughter weight

## Abstract

**Objectives:** The current research aimed to determine the optimal broiler size for consumers using an integrated approach encompassing carcass characteristics, physical properties, nutritional profiles, sensory evaluation, and microbial load in meat.

**Materials and Methods:** A total of 45 broiler birds were chosen and categorized into three groups: 1.0–1.5 kg, 1.5–2.0 kg, and 2.0–2.5 kg. Each group consisted of 15 birds. Carcass characteristics and several physical properties, including drip loss, pH, water-holding capacity, cooking loss, and marinade retention, were measured. The following were assessed during the nutritional analyses: moisture, crude protein, ether extract, and ash. Additionally, microbial loads in meat, including TVC (Total Viable Count) and TCC (Total Coliform Count), were determined.

**Results:** All the carcass characteristics, such as the weight of breast, drumstick, thigh, wings, and dressing percentage, differed significantly among the three weight groups. The 2.0–2.5 kg group achieved the highest dressing percentage (72.42%), eviscerated weight (1.91 ± 0.10 kg), and breast yield (596.58 ± 67.44 gm). The physical and nutritional properties showed non-significant differences among the groups. In the sensory evaluation, broilers weighing 2.0–2.5 kg were preferred over the alternative options. Microbiological studies indicated that the TVC and TCC ranged from 5.54 to 5.89 CFU/gm and 4.56 to 4.95 CFU/gm, respectively, across all categories, indicating acceptable levels for food safety.

**Conclusions:** Broilers weighing 2.0–2.5 kg showed superior carcass yield, consumer appeal, and economic viability.

## 1. Introduction

The poultry industry is mostly driven by consumers and has various sectors to meet their needs [1]. Poultry is becoming increasingly popular day by day due to its high nutritional value and affordable pricing compared to other meats [2]. In Bangladesh, most chickens are sold either live or immediately slaughtered and dressed. The bulk of white meat comes from unorganized wet markets, with only 5–10% coming from traditional processing facilities [3].

The average market weight of a live broiler in Bangladesh ranges from 1 to 3.5 kg [4]. This weight range is very common due to consumers’ behavior and purchase capacity. Usually, consumers prefer to purchase a whole chicken instead of specific retail cuts. Broiler chickens are highly susceptible to hot, humid conditions in Bangladesh. They have difficulty cooling down due to insulating feathers, a lack of sweat glands, and a remarkably high mass-to-body surface area ratio. Instead, they must pant to release heat [5, 6, 7]. A study found that broilers exposed to heat stress had significantly lower body weight gain than those in thermoneutral conditions [8].

Birds of various ages and weights are processed to satisfy the particular requirements of consumers [1]. Changes in meat quality are influenced by several factors, including age of birds, live weight at processing, strain, fasting, cage loading, and transportation [9, 10]. More specifically, from the customer’s perspective, meat quality encompasses factors such as texture, flavor, juiciness, and appearance. The acceptability of a product can be finally determined by these factors, which are influenced by pH, color, tenderness, water holding capacity, and cooking loss [11, 12]. Customers favor chicken meat, especially breast and thigh meat, for its high nutritional content, good taste, affordable cost, and lack of religious constraints [13]. Therefore, it is important to understand how sex, strain, slaughter weight, and age affect meat quality over time so that the industry can optimize yields and avoid downgraded carcasses.

Meat safety and quality have become significant concerns as consumers are now more conscious. With increased broiler body weight, meat characteristics such as appearance, texture, juiciness, firmness, tenderness, odor, water-holding capacity, cooking loss, and flavor are affected [1]. When producing value-added meat products, processors rely heavily on meat’s quantitative properties, including pH, water-holding capacity, drip loss, shear force, cooking loss, collagen content, protein solubility, cohesiveness, fat-binding capacity, and shelf life [14]. A quality feature that affects consumer preference is the color of poultry meat, which varies by species, genotype, age, and muscle type and is influenced by the amount of myoglobin present in the muscle tissue [15].

Consumers favor cost-effective weight categories that supply sufficient meat for family meals. Knowledge of weight preferences allows producers and sellers to tailor production and marketing to consumer needs. While heavier birds might yield more meat, their profitability is determined by their overall economic efficiency. Age and market weight are commonly used to classify broilers, which affects the meat’s texture, nutritional content, and optimal cooking techniques [16]. Broilers raised to a live body weight of 2.1 to 2.2 kg showed improved welfare, immunological status, and meat quality [17]. The meat from younger broilers is softer, while the meat from older birds has a more developed flavor and takes longer to cook [14]. However, the ideal slaughter weights of broilers in relation to consumer benefits across all aspects have not yet been fully investigated. To resolve the situation, it is necessary to explore the proper age and body weight of the broiler for slaughter [17].

Therefore, there is a need to evaluate and have accurate knowledge of carcass characteristics and meat quality by weight category. The main objective of this research is to identify a suitable slaughter age for broilers to achieve the best carcass quality.

## 2. Materials and Methods

### 2.1. Ethical approval

The study protocol was approved by the Animal Experimentation Ethics Committee of Sher-e-Bangla Agricultural University, Dhaka, Bangladesh (Approval No. SAU/AEEC/D-1525-0005).

### 2.2. Study area & sample collection

The research area focused on the different broiler selling points in Dhaka city. Retail markets were randomly selected to collect dressed broiler samples slaughtered using the halal method. A total of 45 birds were randomly selected for this study into three groups, namely Group A, B, and C, with 15 broilers in each group. The average body weight was 1.0–1.5 kg in Group A, 1.5–2.0 kg in Group B, and 2.0–2.5 kg in Group C.

### 2.3. Determination of gross characteristics of the broiler carcass

Live weight was recorded by using an electronic weighing balance. Carcass appearance and presence of defects were evaluated using a scale of five [18]. The color of breast muscle is graded by using the DSM Broiler Color Fan. Dressed weight was considered as hot (eviscerated) carcass weight measured without skin.

The carcasses were segmented into specific parts, including breast, thigh, drumstick, and wings. Each of these cut-up parts was weighed and recorded individually. The head, neck, feet, back, liver, heart, gizzard, and giblets were also weighed and recorded individually. The carcasses were carefully dissected to isolate the abdominal fat pad and separated from other tissues and organs. The weight of the offal, feathers, and blood was also measured and recorded separately.

### 2.4. Physical characterization of the broiler carcass

Physical characteristics of broiler carcasses were assessed on the same day as sample collection. Samples not immediately analyzed were stored at 4°C for short-term refrigeration. The pH of broiler meat was measured using a digital pH meter (Hanna HI-98107) according to the manufacturer’s instructions. Drip loss [19], water holding capacity [20], cooking yield/loss [21], and marinade retention [22] were measured as described previously. Briefly, for measuring drip loss, the muscle was placed in a polythene bag, securely sealed with thread, and hung in a refrigerator set at 4°C for 24 h.

Afterward, those were removed from the bags and reweighed immediately to record their final weights. The water-holding capacity was calculated as the difference between the initial and final weights under external pressure. To determine the cooking yield, meat samples were rested at 4°C for 24 h, then samples of 50 gm were cooked at 80°C for 45 min. After cooking, the samples were reweighed. The fillets were weighed to determine their initial weight for assessing marinade retention. A 4% brine solution was prepared with the recommended salt concentration to achieve the desired marinade strength. The fillets were immersed in the marinade solution, ensuring thorough coverage, and tumbled for 15 min to facilitate marinade absorption. After tumbling, the fillets were removed from the marinade and weighed again to measure their weight after marinade uptake. The final weight of the fillets was measured after holding overnight to determine the marinade retention percentage.

### 2.5. Nutritional profile of broiler meat

Samples were prepared by grinding to achieve a homogeneous consistency, ensuring accuracy in subsequent measurements. Samples not immediately analyzed were stored under short-term refrigeration (2–3 days) at 4°C to maintain their integrity. The moisture content was determined by oven drying. The Kjeldahl method was used to quantify nitrogen content, which was subsequently converted to protein content. The Soxhlet extraction method was used to determine the crude fat and ether extract (EE%) content. Ash content was determined by incinerating the samples in a muffle furnace at 550°C. These procedures were followed according to AOAC (1990) guidelines.

### 2.6. Microbiological analysis

The microbiological analyses, TVC (Total Viable Count) and TCC (Total Coliform Count), were performed according to Drebes et al. [23] and the protocols provided by the 3M Petrifilm supplier (TM Petrifilm). Briefly, 25 gm of broiler breast meat was taken using a sterile scalpel and placed into individual stomacher bags. A homogenized suspension will be made by placing 225 ml of 0.1% buffered peptone water into the bag containing the meat sample. 1 ml of each dilution was dispensed onto the center of the 3M Petrifilm plate using a sterile pipette. A plastic spreader or a flat, sterile object was used to evenly distribute the sample across the film, ensuring that no bubbles were introduced during this step. The inoculated Petrifilm plates were incubated for 24 h at 35°C. The microbial colonies were counted and calculated to determine the colony-forming unit. To express the population in colony-forming units per gram (CFU/gm), the number of typical colonies is multiplied by the inverse of the dilution factor.

### 2.7. Sensory evaluation

Sensory characteristics were assessed using a five-point categorical scale as described previously with minor modifications [24]. Briefly, unseasoned broiler breast meat was cooked in a prewarmed 100°C electric oven to a final core temperature of 70°C. Samples were removed from the oven, tempered for 15 min in the pan, and prepared for sensory analysis by cutting a 2-cm-wide strip parallel to the fibers. This strip was then cut into 2 or 3 cubes of 2 cm. Thereafter, equal bite-sized portions of cooked meat samples were coded and served at room temperature (25°C) to 16 trained taste panelists composed of 10 faculty members and 6 research fellows. The panelists received each sample separately, with water and bread provided as neutralizers between samples. A five-point category scale was used to evaluate the sensory characteristics of the products as follows: Color, off-odor, juiciness, flavor intensity, flavor-liking, and Overall acceptability. The scorecard (scale 1 to 5) used to evaluate sensory attributes ranges from the lowest intensity (score 1) to the highest (score 5).

### 2.8. Data analysis

The data were expressed as mean ± SD, and analyzed by one-way ANOVA, with carcass weight at slaughter as the main factor, using SPSS Ver. 24 (IBM SPSS, 2016). The different means were compared using the Duncan multiple-range test (1955) at the 95% confidence level.

## 3. Results and Discussion

### 3.1. Gross characteristics of broiler carcasses at different slaughter weights

**Figure 1.**
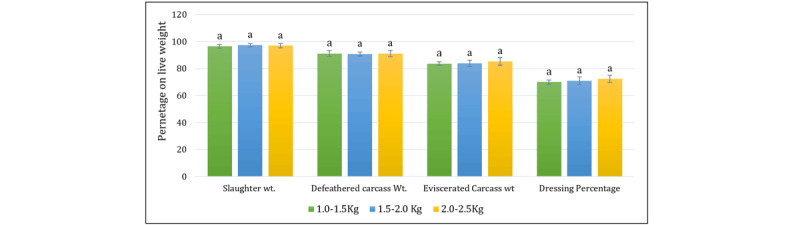
Gross characteristics of the broiler carcass are based on live weight. Bars with the same letter (a) do not differ significantly (*p* > 0.05).

**Figure 2.**
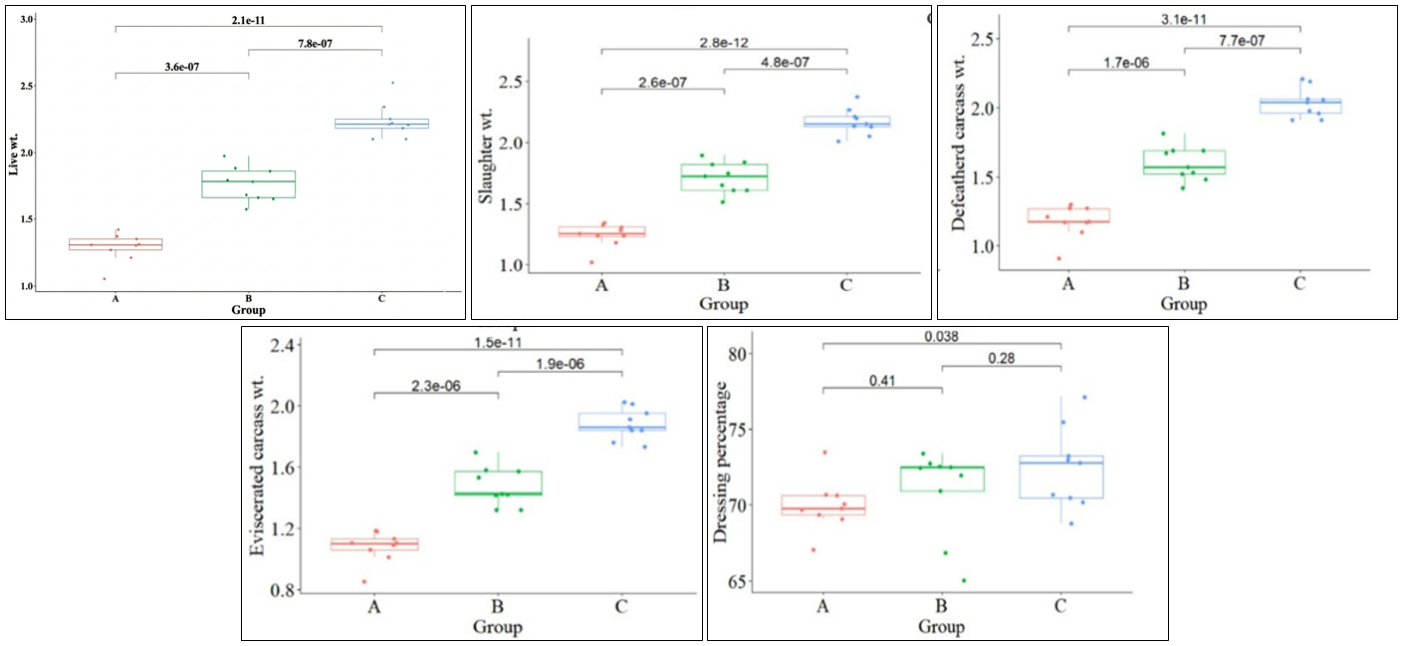
Gross characteristics of broiler carcasses in different groups. A = (1.0–1.5 kg), B = (1.5–2.0 kg), C = (2.0–2.5 kg). Different carcass weights are expressed in (kg).

The gross characteristics of the broiler carcass, as shown in Figures 1 and 2, indicate that the dressing percentages improved gradually across groups, from 69.97% in Group A to 72.42% in Group C. It is clear that the dressing percentage increases with live weight and age, in line with earlier studies by Hussein et al. [2] and Baéza et al. [25]. Further, Group C demonstrated improvements in breast yield, dressing percentage, and meat quality, which align with previous studies [17, 26]. In addition, Park et al. [27] reported that water-holding capacity, cooking yield, and protein content increased with slaughter age. Therefore, it is possible to assume that a higher dressing percentage indicates higher meat quality in broilers from Group C.

Live weight, slaughter weight, defeathered carcass weight, and eviscerated carcass weight were increased steadily (*p* < 0.05) from the lower weight group (A) to the higher weight group (C). Furthermore, the weight of the parameters from live weight to eviscerated carcass weight was reduced homogenously. This reduction is due to the removal of non-edible parts, including blood, feathers, head, shanks, and viscera. Such consistency indicates efficient processing across weight groups. Higher defeathered yields arising from better genetics and feeding practices [28]. However, these findings on meat quality enhancement partially disagree with those reported by Hussein et al. [2] and Ikusika et al. [29], who found that increased slaughter weight increased meat yield but did not affect meat quality. The eviscerated carcass weight not only depends on bird size but may also depend on age and genetic strain [2]. These findings are important because they help balance economic returns and meat quality in broiler live weight.

**Figure 3.**
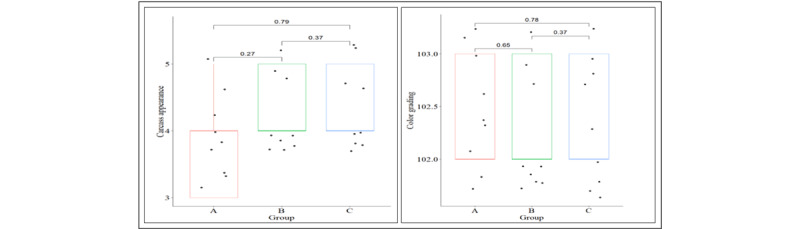
Appearance and color grading of broiler carcass. A = (1.0–1.5kg), B = (1.5–2.0kg), C = (2.0–2.5kg).

The carcass appearance (scored 1–5) and color grading (101–108) were similar across weight categories. Carcass appearance was primarily scored with a mode value of 4, indicating similar visual quality of broilers across live weight groups (Figure 3). Hence, uniformity suggests that non-detrimental live weight variations are associated with better carcass visual quality in broilers. This is due to the nationwide corn-based diet. Increased slaughter weight has been shown to improve carcass aesthetic quality, including color parameters [29, 30].

However, this study disagreed because it compared the meat of male and female broilers across different strains. Additionally, their feeding practice differed from that in this study. After slaughter, the carcass color may vary over time, as reported in another study [31]. Consequently, the consistent outcome of this investigation indicated that all samples were examined simultaneously.

### 3.2. Characteristics of major cuts of broiler carcass

**Figure 4.**
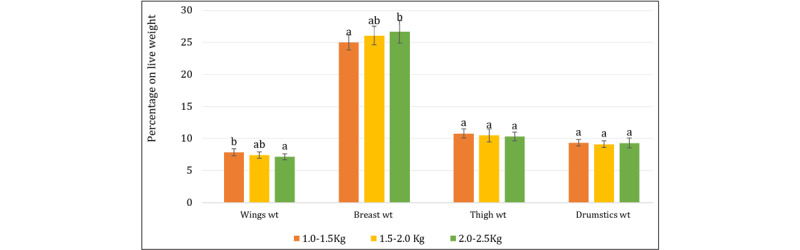
Major Cuts of Broiler Percentage on Live weight basis. Bars with different letters (a, b, c) above them indicate significant differences (*p* < 0.05).

**Figure 5.**
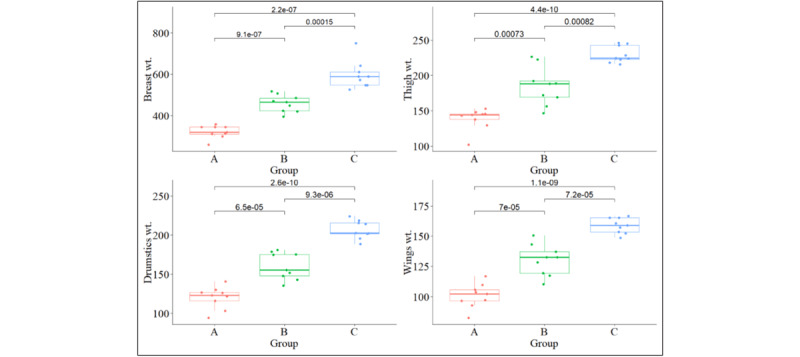
Major Cuts of Broiler in Different Groups. A = (1.0–1.5kg), B = (1.5–2.0kg), C = (2.0–2.5kg). Different carcass weights are expressed in (kg).

The major differences in carcass cuts across weight categories are breast, wings, thighs, and drumsticks (Figures 4 and 5). Breast weight directly correlates with meat yield, a primary revenue source for poultry producers [32], and is associated with increased live weight (LW). These findings were also reported by Mudalal and Zaazaa [33] in relation to improving breast yield at optimal slaughter weight, along with a presumable indicator of higher protein and lower PUFA levels in heavier broilers [34]. Similar to our study, Baéza et al. [35] and Marpana et al. [36] found that a broiler weight of 2.0–2.5 kg would yield higher breast meat and provide nutritional and economic viability in broiler production. Wing weight increased proportionally with live weight. The wings’ weight as a percentage of live weight decreased from 7.83% in Group A to 7.12% in Group C, showing that larger birds allocate less weight to their wings. However, Lawlor et al. [37] found that wing proportions are invariant with weight. This dissimilarity between studies may be due to the strain differences. The thigh and drumstick weights increased with slaughter weight. Things usually accounted for between 10.3% and 10.77%, and drumsticks between 9.1% and 9.3%. Therefore, a significant portion of premium cuts, including the breast and thighs, are found in heavy broilers [38]. This suggests that selecting for higher live weights can improve the yield of valuable meat cuts.

### 3.3. Characteristics of non-preferred cuts of broiler

**Figure 6.**
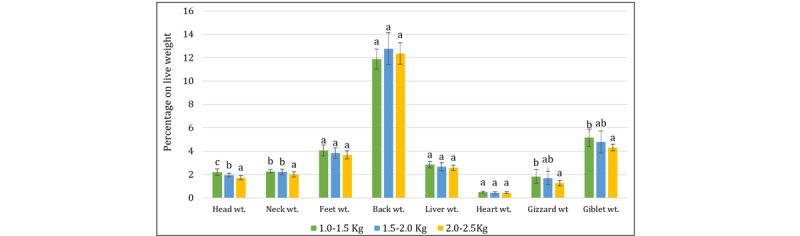
Non-preferred cuts of broiler on a live weight basis. Bars with different letters (a, b, c) above them indicate significant differences (*p* < 0.05).

The association of non-preferred cuts along with organ weights in broiler carcasses as influenced by live weight categories is presented in Figure 6. The head and neck weights increased with live weight; however, they decreased slightly when expressed as a percentage of live weight (Figure 6). This is probably because these parts grow substantially more slowly than other parts of the body [37]. The proportional weights of the head, neck, feet, back, liver, heart, and giblets all increased as the live weight of the broiler increased, proving that larger broilers produce more developed by-products. This is because the organs’ metabolic and functional significance demonstrates their unique qualities. Increased liver and heart weight showed a direct correlation with body size, as demonstrated by Mehmet et al. [39] and Gök and Tolun [40]. Gizzard weight, however, did not differ significantly between groups because variations in live weight tended to reduce its variability.

### 3.4. Characteristics of the inedible parts of the broiler carcass

**Figure 7.**
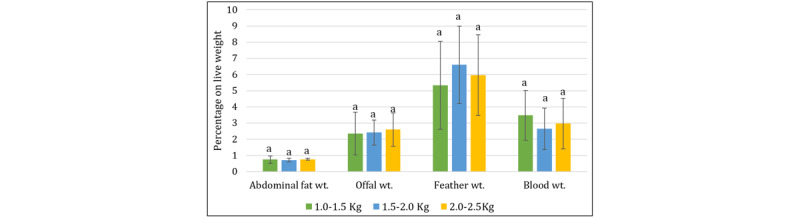
Inedible parts percentage on a live weight basis. Bars with the same letter (a) do not differ significantly (*p* > 0.05).

Abdominal fat increased slightly (only 0.72%–0.77% of LW) according to broiler slaughter weight (Figure 7). This measure correlates with the results of Abo Ghanima et al. [41]. Moreover, live weight considerably raised feather weight from 66.44 gm to 133.33 gm, while there was no discernible difference in terms of percentage (5.33%–6.60%) (*p* > 0.05).

However, the weight of the offal and blood did not change, either on a weight or percentage basis. This may indicate that the inedible components stay relatively stable regardless of the birds’ weight and age.

### 3.5. Physical characteristics of the broiler carcass

Various quality parameters of broiler meat within the slaughter weight range are shown in Table 1. These are fundamental characteristics for assessing meat quality, including appeal, texture, stability, and further processing. All these affect consumer satisfaction and processing efficiency.

**Table 1.** Quality properties of Broiler meat.

Parameter	Market weight (kg)		
	1.0–1.5	1.5–2.0	2.0–2.5
pH	6.13^a^ ± 0.16	6.19^a^ ± 0.13	6.19^a^ ± 0.13
Drip loss %	3.26^a^ ± 1.91	2.90^a^ ± 1.11	3.22^a^ ± 0.69
Water holding capacity%	22.42^a^ ± 4.34	21.44^a^ ± 6.57	23.81^a^ ± 8.64
Cooking Yield%	85.11^a^ ± 4.20	84.71^a^ ± 1.48	84.70^a^ ± 1.53
Cooking Loss%	14.89^a^ ± 4.20	15.29^a^ ± 1.48	15.30^a^ ± 1.53
Marinade retention %	2.11^a^ ± 0.58	2.26^a^ ± 1.34	1.72^a^ ± 0.51

The different superscripts among the three groups within a row indicate significant differences (*p* < 0.05). SD = standard deviation

The normal pH range is 5.8–6.2 and is typically associated with high-quality meat. This study showed the pH values within the normal range across all weight categories: 6.13, 6.19, and 6.19, respectively. The pH is a critical indicator of broiler meat quality and is altered by various factors such as genetics and pre- and post-slaughter handling [42]. pH levels are among the crucial factors that influence meat tenderness, juiciness, and microbial stability. Thus, this study indicates that the pH of broiler meat is relatively stable and is affected only slightly by slaughter weight, suggesting similar microbial stability across weight categories (Groups A, B, and C). Additionally, stress-free handling and transport has been ensured for all birds.

The greatest amount of drip loss occurred in Group A at 3.26% and decreased to 2.90% in Group B before rising again to 3.22% in Group C (2.0 – < 2.5 kg category). According to Yaranoğlu et al. [43], reduced drip loss also implies higher water-holding capacity and, consequently, reduced economic loss due to weight loss during storage, thereby enhancing juiciness and texture. The lower drip loss in Group B suggests that this weight range may be optimal for moisture retention, thereby potentially increasing the meat’s sensory appeal and shelf life. Such moderate slaughter weights (around 2.0 kg) suggest a favorable compromise between moisture retention and economic loss, coupled with good meat quality.

The water-holding capacity varied slightly across the weights, ranging from 22.42% to 23.81%. Hence, it can be assumed that a bigger bird would retain more moisture, as observed in Group C. In a previous study, Samuel et al. [44] got a wide range of water-holding capacity in broiler breast meat with a mean of 16.7%. Factors influencing WHC include declining pH, protein oxidation, and changes in muscle cell structure, particularly in pale, soft, and exudative meat. According to Park et al. [27], increased WHC enhances juiciness in meat and reduces cooking losses, thereby satisfying consumers. The increased WHC in the heaviest broilers may make their meat more desirable in markets [45]. This highlights the importance of selecting heavier birds to enhance both meat quality and market appeal.

Cooking yield is the proportion of meat retained after cooking, whereas cooking loss estimates moisture losses throughout the cooking process. All cooking yields across categories ranged from 84.70% to 85.11%, indicating that slaughter weight has little effect on this quality parameter. On the other hand, cooking loss ranged from 14.89% to 15.30%, with no significant difference across weight categories. Slaughter weight alone had no significant effect on cooking yield or loss. The minimum amount of water that the meat can contain is determined by the physiological condition of the bird at the time of slaughter [46]. Processing factors largely affect WHC, muscle structure, and protein denaturation levels in chicken meat, which, in turn, affect cooking yield or loss [47]. Besides, chicken handling both before and after slaughter, as well as certain cooking methods, endpoint temperatures, and cooking times, have a substantial impact on moisture retention and overall yield [47, 48]. However, this study showed that cooking performance remained unchanged across varying broiler weight categories.

Marinade retention was 2.26% in Group B, followed by 2.11% and 1.72% in Group A and Group B, respectively (*p* > 0.05). The higher marinade retention in the Group B category indicates that this weight range was likely ideal for optimal flavor absorption during marination and could thus be preferred for processed meat products. Therefore, results indicate that broilers in Group B are more potential candidates for marinated products.

### 3.6. Nutritional profiles of broiler meat in different slaughter weight categories

The nutritional composition of broiler meat is shown in Table 2. The compositions were not different across age groups (*p* > 0.05). Moisture content plays a significant role in meat juiciness and texture. The results showed that moisture content stood at 74.71%, 76.10%, and 74.91% in the 1st, 2nd, and 3rd weight groups, respectively. There is a slight increase in moisture content in Group B, indicating more tender meat than in the other two groups. This is consistent with studies by Zanetti et al. [34], which found that meat moisture content is affected by both slaughter age and body weight, thereby influencing its texture and sensory appeal. The dry matter content of the chicken was 25.30% in Group A, 23.90% in Group B, and 25.09% in Group C.

**Table 2.** Nutritional profiles of broiler meat.

Parameter (%)	Market weight (kg) (Mean ± SD)		
	1.0–1.5	1.5–2.0	2.0–2.5
Moisture	74.71^a^ ± 0.06	76.10^b^ ± 0.49	74.91^a^ ± 0.23
CP (DM basis)	92.46^a^ ± 0.07	90.60^a^ ± 1.53	91.69^a^ ± 1.02
EE (DM basis)	0.98^a^ ± 0.24	1.48^a^ ± 0.21	1.18^a^ ± 0.01
Total Ash (DM basis)	4.79^a^ ± 0.10	5.17^a^ ± 0.51	5.12^a^ ± 0.82

The different superscripts among the three groups indicated significant differences (*p* < 0.05). (CP = crude protein; EE: Ether extract; SD = standard deviation).

The crude protein content did not vary across weight groups of broiler chickens; levels were 92.46%, 90.60%, and 91.69% for the lower, middle, and higher weight groups, respectively (*p* > 0.05). Studies show that the protein content of broiler meat increases with slaughter age, with older birds having higher protein levels in breast or thigh meat [27]. Thus, the weight of the bird and its protein content show a positive correlation, although this was not observed in the current study.

The ether extract (fat) levels of broiler meat were 0.98%, 1.48%, and 1.18% across the three weight categories (*p* > 0.05). However, the second group (1.5–<2.0 kg) had the highest fat content, which presumably favors better flavor and palatability. The higher fat content in this weight category indicated that these birds were very close to reaching the optimal fattening stage for their size. Total ash levels, representing the inorganic mineral content, were 4.79%, 5.17%, and 5.12% in the weight categories. It was highest in the 1.5–<2 kg group, suggesting that animals may have a marginally richer mineral profile, which could improve mineral intake. This trend aligns with that of Lawlor et al. [37], who found that mineral content in broiler meat increased with bird size. The small variation in the ash content among all weight categories may have been achieved through standard feeding and genetic management practices, making broiler meat consistent in its mineral content.

### 3.7. Microbiological analysis of broiler meat

The microbial analysis of broiler meat samples reveals substantial differences in contamination, underscoring the need for improved hygiene and processing standards. Total Viable Count (TVC) and Total Coliform Count (TCC) were presented in Table 3. High TVC values, ranging from log_10_ 5.89 ± 1.05 to log_10_ 5.54 ± 0.65 CFU/gm, are consistent with findings by Julqarnain et al. [49], which emphasize the effect of microbial loads on meat quality, sensory characteristics, and shelf life. Likewise, TCC with values between log_10_ 4.95 ± 0.42 and log_10_ 4.56 ± 0.60 CFU/gm further supports this issue [49]. Thames et al. [50] found that microbial loads, including aerobic bacteria, coliforms, and lactic acid bacteria, significantly decreased after processing, especially after chilling, thereby enhancing shelf life and sensory quality. Together, these findings underscore the urgent need for strict sanitary practices, appropriate storage conditions, and rigorous adherence to food safety standards to minimize microbial contamination, enhance meat quality, and ensure consumer safety.

**Table 3.** Microbial load (TVC) in broiler meat.

Parameters	Market weight category of Broiler (Mean ± SD)		
	1.0–1.5 kg	1.5–2.0 kg	2.0–2.5 kg
TVC (CFU/gm)	5.54^a^ ± 0.65	5.89^a^ ± 1.05	5.72^a^ ± 0.82
TCC(CFU/gm)	4.95^a^ ± 0.42	4.71^a^ ± 0.45	4.56^a^ ± 0.60

The different superscripts among the three groups indicated significant differences (*p* < 0.05). (TVC = total viable count; TCC = total coliform count; SD = standard deviation).

### 3.8. Sensory evaluation of broiler meat

The sensory evaluation of broiler meat, summarized in Table 4, indicates considerable variation among weight categories. Among the evaluated parameters, weight categories A (1.0–1.5 kg) and C (2.0–2.5 kg) showed more balanced sensory attributes than the medium-weight group B (1.5–2.0 kg). Heavier broiler meat is typically juicier and more tender, while lighter meat tends to be harder, more cohesive, and chewier [51]. Juiciness in breast meat is more closely associated with water-holding capacity, which is often better in meat from heavier birds. This is attributed to higher fat content and moisture release. Besides, enhanced juiciness and texture of meat from heavy birds (> 2.0 kg) contribute to higher overall palatability scores in processed products such as nuggets [52, 53]. A strong meat flavor is often considered a desirable quality attribute in sensory analysis. In this study, it was observed that the heavy bird category is typically perceived by consumers as natural, wholesome, and indicative of high-quality meat. However, the lighter group (1.0–1.5 kg) showed a sensory attribute pattern similar to the heavy bird group. This discrepancy may be driven by panelists not consistently perceiving minor differences, given minimal variation among chicken weight groups.

**Table 4.** Sensory evaluation parameter of broiler meat.

Parameter	Market weight (Kg)		
	1.0–1.5 kg	1.5–2.0 kg	2.0–2.5 kg
Color	3 (Intermediate)	2 (Pale)	3 (Intermediate)
Off-odor	1 (Very weak)	1 (Very weak)	1 (Very weak)
Juiciness	3 (Intermediate)	2 (Juicy)	3 (Intermediate)
Flavor intensity	3 (Intermediate)	3 (Intermediate)	4 (Strong)
Flavor-liking	3 (Intermediate)	2 (Like)	2 (Like)
Overall acceptability	3 (Intermediate)	3 (Intermediate)	2 (Like)

The data represent the mean value obtained on a scale of 1–5.

### 3.9. Correlation study

**Figure 8.**
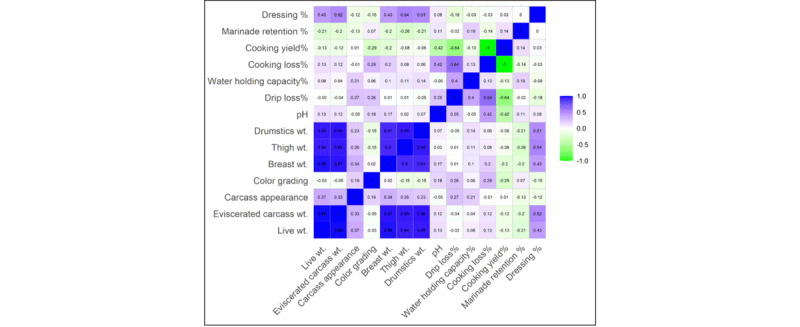
Correlation matrix among physical quality parameters and the weight of different body parts of the carcass.

Correlation analysis indicates that “Live wt.” has a weak positive correlation with “Cooking loss%” (*r* = 0.13) and weak negative correlations with “Cooking yield%” (*r* = –0.13) and “Drip loss%” (*r* = – 0.02) (Figure 8). This suggests that heavier birds may lose more moisture and nutrients during cooking. Therefore, “Live wt.” alone will not be an optimal indicator of post-cooking losses. Notably, “Drip loss %” demonstrates a moderate positive correlation with “Cooking loss %” (*r* = 0.64), indicating that chicken meat with higher fluid loss during storage also tends to exhibit more significant quality loss during thermal processing [54]. In contrast, there is a negative correlation with “Cooking yield %” (*r* = – 0.64). An inverse correlation was observed between “Cooking loss %” and “Cooking yield %” (*r* = –1), reflecting the direct relationship between mass and final product output. These findings suggest that lower drip and cooking losses may lead to achieving maximum cooking yield. Maximum water-holding capacity is essential for improving the quality of chicken meat [55].

The relationships with pH, “Cooking loss %,” and “Cooking yield %” exhibit moderate positive and negative correlations, respectively. This indicates that, with the pH raised, the extent of weight loss during cooking was also increased. This might be because of greater exudation of water and soluble nutrients during heat treatment. In contrast, the negative correlation with “Cooking yield %” (*r* = –0.42) indicates that higher pH values are associated with lower final product yield. Very low pH can be associated with low water-holding capacity, which can also lead to increased cooking loss [14]. Managing optimal pH during slaughter handling, chilling practices, and processing conditions is therefore critical to reducing cooking loss and maximizing cooking yield from chicken meat.

The correlation among carcass appearance, color grading, live weight, and drip loss shows some valuable findings. A positive correlation (*r* = 0.37) was observed between live weight and carcass appearance, indicating that those with greater body weight possess more developed and more appealing carcasses. Surprisingly, a positive correlation was also observed between drip loss and carcass appearance (*r* = 0.27), with the more highly graded carcasses losing more water in storage. But there is still a contradiction in that the “carcass appearance” depends upon the dietary supplement, sex, age, and strain [56, 57].

Water-holding capacity was positively correlated with drip loss (*r* = 0.4), indicating that meat samples with higher water-holding capacity tend to release more fluid during storage [58] and incur greater economic losses [43]. Conversely, marinade retention was weakly negatively correlated with drip loss (*r* = –0.02), suggesting that meat samples with externally added ingredients tend to lose less moisture over time. This negative association highlights the role of marinade functionality in improving the quality of chicken meat [59, 60, 61].

**Figure 9.**
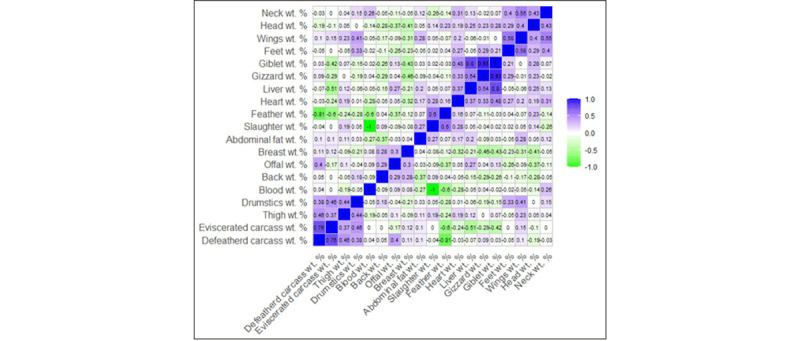
Correlation among the percentage of different body parts of the carcass based on live weight.

In Figure 9, A significant parameter, “Breast wt.%,” has a moderate negative correlation with the non-preferred cuts of the carcass (e.g., Neck wt.%, Head wt.%, Wings wt.%, Feet wt.%, Giblet wt.%, Gizzard wt.%, Liver wt.%, and Heart wt.%). These findings align with [62, 63]. This study clearly shows that as breast weight increases, the non-preferred cut tends to gain less weight in broiler chickens. Another notable finding is that “Drumstick wt.%” is positively correlated with “Wings wt.%” (*r* = 0.33). Indicating that a larger chicken will gain more weight in both drumsticks and wings. A customer who needs these portions of the chicken can focus on the chicken’s live weight.

In addition, “Slaughter wt.%” positively correlates with “Abdominal fat wt.%” (*r* = 0.27). For white meat, most people prefer lean cuts. Many customers discard this portion who do not like it. Heavier birds develop abdominal fat as their body weight increases. In this case, lower slaughter weight can be a criterion for selecting leaner meat.

## 4. Conclusions

This study confirms that slaughter weight plays a critical role in determining broiler carcass characteristics and overall meat quality. Birds in the highest weight category demonstrated better yield and consumer appeal. While live weight is not a reliable predictor of post-cooking meat quality, processing-related indicators—especially drip loss and meat pH—are crucial determinants of the final product’s quality. Although minor declines in certain quality attributes were observed, the overall meat retained desirable processing and consumer satisfaction characteristics. Additionally, higher slaughter weight was associated with increased abdominal fat deposition, indicating a concurrent increase in yield that should be considered in production decisions. These insights support the producers and sellers in supplying the right products, improving efficiency, and meeting consumer demand more effectively. Therefore, more research is needed to clarify the weight-based meat quality while accounting for a wide range of bird populations and various pre- and post-slaughter conditions.

## Data Availability

The data presented in this study are available from the corresponding author upon reasonable request.

## List of abbreviations

kg, kilogram; %, percent; gm, gram; TVC, total viable count; TCC, total coliform count; CFU, colony forming unit; °C, degree Celsius; ml, milliliter; cm, centimeter; CP, crude protein; DM, dry matter; EE, ether extract; SD, standard deviation; LW, liveweight; PUFA, polyunsaturated fatty acid; WHC, water holding capacity; wt., weight.
